# Supplementary material for: Untargeted metabolites profiling of volatile components of Chinese Antique Lotus (Nelumbo nucifera Gaertn.) using solid-phase microextraction (SPME) GC/MS
Source: PeerJ. 2025 Jun 19;13:e19600. doi: 10.7717/peerj.19600 (PMC12182725; doi:10.7717/peerj.19600)
Supplement: Supplemental Information 1 — Note: RI (exp.): Experimental retention indices; RI (lit.): literature retention indices (PubChem, NIST, and the Pherobase); C1-C64 is the code for each compound. When graphing, we use specific designations for the corresponding compounds. [file peerj-13-19600-s001.docx]

| Table S1. Identification of different types of compounds. | | | | | | | | |
| --- | --- | --- | --- | --- | --- | --- | --- | --- |
| NO. | Compounds type | RT | Compound | CAS | | RI(lit.) | | RI(exp.) |
| Terpenoids (Monoterpenoids) | | | | | | | | |
| C1 | C10H16 | 6.856 | 3-Thujene | | 2867-05-2 | | 929 | 924 |
| C2 | C10H16 | 7.082 | α-Pinene | | 7785-70-8 | | 937 | 930 |
| C3 | C10H16 | 7.586 | Camphene | | 79-92-5 | | 952 | 944 |
| C4 | C10H16 | 8.511 | Sabinene | | 3387-41-5 | | 974 | 970 |
| C5 | C10H16 | 8.615 | β-Pinene | | 127-91-3 | | 979 | 973 |
| C6 | C10H16 | 9.236 | β-Myrcene | | 123-35-3 | | 991 | 990 |
| C7 | C10H16 | 9.268 | 2-Carene | | — | | 991 | 991 |
| C8 | C10H16 | 9.708 | α-Phellandrene | | 99-83-2 | | 1005 | 1003 |
| C9 | C10H16 | 10.213 | α-Terpinene | | 99-86-5 | | 1017 | 1015 |
| C10 | C10H16 | 10.704 | Limonene | | 5989-27-5 | | 1030 | 1026 |
| C11 | C10H18O | 10.788 | Eucalyptol | | 470-82-6 | | 1032 | 1028 |
| C12 | C10H16 | 11.597 | β-*cis*-Ocimene | | 3338-55-4 | | 1038 | 1046 |
| C13 | C10H16 | 12.011 | γ-Terpinene | | 99-85-4 | | 1060 | 1056 |
| C14 | C10H16 | 13.298 | Terpinolene | | 586-62-9 | | 1088 | 1086 |
| C15 | C10H18O | 13.699 | *cis*-2-p-Menthen-1-ol | | 29803-82-5 | | 1122 | 1095 |
| C16 | C10H18O | 17.27 | Terpinen-4-ol | | 562-74-3 | | 1177 | 1174 |
| C17 | C10H18O | 17.885 | α-Terpineol | | 98-55-5 | | 1189 | 1187 |
| C18 | C10H18O | 18.603 | α-Cyclogeraniol | | 6627-74-3 | | 1184 | 1203 |
| Terpenoids (Sesquiterpenoids) | | | | | | | | |
| C19 | C15H24 | 25.99 | Copaene | | 3856-25-5 | | 1376 | 1372 |
| C20 | C15H24 | 26.708 | β-Elemene | | 515-13-9 | | 1391 | 1389 |
| C21 | C15H24 | 27.833 | Caryophyllene | | 87-44-5 | | 1419 | 1417 |
| C22 | C15H24 | 28.163 | *cis*-β-Farnesene | | 28973-97-9 | | 1444 | 1425 |
| Table S1 (*continued*) | | | | | | | | |
| NO. | Compounds type | RT | Compound | | CAS | | RI(lit.) | RI(exp.) |
| C23 | C15H24 | 29.192 | Humulene | | 6753-98-6 | | 1454 | 1450 |
| C24 | C15H24 | 29.45 | (*E*)-β-Famesene | | 18794-84-8 | | 1457 | 1457 |
| C25 | C15H24 | 29.632 | γ-Gurjunene | | 22567-17-5 | | 1473 | 1461 |
| C26 | C15H24 | 30.168 | γ-Muurolene | | 30021-74-0 | | 1477 | 1474 |
| C27 | C15H24 | 30.317 | Germacrene D | | 23986-74-5 | | 1481 | 1478 |
| C28 | C15H24 | 30.945 | Bicyclogermacrene | | 24703-35-3 | | 1495 | 1494 |
| C29 | C15H24 | 31.061 | δ-Selinene | | 28624-23-9 | | 1493 | 1496 |
| C30 | C15H24 | 31.391 | δ-Guaiene | | 3691-11-0 | | 1505 | 1505 |
| C31 | C15H24 | 31.656 | γ-Cadinene | | 39029-41-9 | | 1513 | 1512 |
| C32 | C15H24 | 32.044 | δ-Cadinene | | 483-76-1 | | 1524 | 1522 |
| C33 | C15H24 | 32.562 | α-Cadinene | | 24406-05-1 | | 1538 | 1535 |
| C34 | C15H24O | 34.27 | Caryophyllene oxide | | 1139-30-6 | | 1581 | 1580 |
| C35 | C15H26O | 36.133 | γ-Eudesmol | | 1209-71-8 | | 1631 | 1629 |
| C36 | C15H26O | 36.475 | α-epi-Cadinol | | 5937-11-1 | | 1640 | 1639 |
| C37 | C15H26O | 36.902 | α-Eudesmol | | 473-16-5 | | 1653 | 1650 |
| C38 | C15H26O | 36.98 | α-Cadinol | | 481-34-5 | | 1653 | 1652 |
| Benzenoids/phenylpropanoids | | | | | | | | |
| C39 | C10H14 | 10.536 | o-Cymene | | 527-84-4 | | 1022 | 1022 |
| C40 | C8H10O2 | 16.765 | 1,4-Dimethoxybenzene | | 150-78-7 | | 1168 | 1163 |
| Fatty acid derivatives | | | | | | | | |
| C41 | C7H14O2 | 4.63 | Butanoic acid, 2-methyl-, ethyl ester | | 7452-79-1 | | 849 | — |
| C42 | C6H14O | 5.096 | 1-Hexanol | | 111-27-3 | | 868 | — |
| C43 | C7H14O2 | 5.322 | 1-Butanol, 3-methyl-, acetate | | — | | — | — |
| C44 | C6H12O3 | 5.775 | Butyric acid, 2-hydroxy-3-methyl-, methyl ester | | 17417-00-4 | | 889 | — |
| Table S1 (*continued*) | | | | | | | | |
| NO. | Compounds type | RT | Compound | | CAS | | RI(lit.) | RI(exp.) |
| C45 | C9H20 | 5.969 | Nonane | | 111-84-2 | | 900 | — |
| C46 | C11H24 | 13.861 | Undecane | | 1120-21-4 | | 1100 | 1099 |
| C47 | C12H26 | 18.389 | Dodecane | | 112-40-3 | | 1200 | 1199 |
| C48 | C11H18O2 | 19.942 | 1-Cyclohexene-1-carboxylic acid, 2,6,6-trimethyl-, methyl ester | | 49815-58-9 | | 1224 | 1233 |
| C49 | C13H26 | 22.283 | (6*E*)-6-Tridecene | | 6434-76-0 | | 1277 | 1286 |
| C50 | C13H28 | 22.84 | Tridecane | | 629-50-5 | | 1300 | 1298 |
| C51 | C14H28 | 26.559 | (*E*)-4-Tetradecene | | 41446-78-0 | | 1379 | 1386 |
| C52 | C14H28 | 26.785 | 1-Tetradecene | | 1120-36-1 | | 1392 | 1391 |
| C53 | C11H16O | 26.986 | Jasmone | | 488-10-8 | | 1394 | 1396 |
| C54 | C14H30 | 27.115 | Tetradecane | | 629-59-4 | | 1400 | 1399 |
| C55 | C15H30 | 30.641 | 1-Pentadecene | | 13360-61-7 | | 1492 | 1486 |
| C56 | C15H32 | 31.191 | Pentadecane | | 629-62-9 | | 1500 | 1500 |
| C57 | C16H32 | 34.237 | (*Z*)-7-Hexadecene | | 35507-09-6 | | 1566 | 1579 |
| C58 | C16H32 | 34.535 | (*Z*)-3-Hexadecene | | 34303-81-6 | | 1587 | 1587 |
| C59 | C16H32 | 34.768 | Cetene | | 629-73-2 | | 1592 | 1593 |
| C60 | C16H34 | 35.052 | Hexadecane | | 544-76-3 | | 1600 | 1600 |
| C61 | C17H32 | 37.646 | 6,9-Heptadecadiene | | 81265-03-4 | | 1667 | 1671 |
| C62 | C14H30O | 37.911 | 1-Tetradecanol | | 2579-04-6 | | — | 1678 |
| C63 | C17H34 | 38.254 | 8-Heptadecene | | 6765-39-5 | | 1692 | 1687 |
| C64 | C17H36 | 38.733 | Heptadecane | | 629-78-7 | | 1700 | 1700 |
| Note: RI (exp.): Experimental retention indices; RI (lit.): literature retention indices (PubChem, NIST, and the Pherobase); C1-C64 is the code for each compound. When graphing, we use specific designations for the corresponding compounds. | | | | | | | | |
