# Supplementary material for: Untargeted metabolites profiling of volatile components of Chinese Antique Lotus (Nelumbo nucifera Gaertn.) using solid-phase microextraction (SPME) GC/MS
Source: PeerJ. 2025 Jun 19;13:e19600. doi: 10.7717/peerj.19600 (PMC12182725; doi:10.7717/peerj.19600)
Supplement: Supplemental Information 2 — Note: we use abbreviations instead of ancient lotus species names, e.g. ZNH stands for N. nucifera ‘Zhongnanhai Antique’ (this nomenclature rule has been mentioned in the main text). “ -” means not detected. [file peerj-13-19600-s002.docx]

| Table S2. Compound content in each tissue of antique lotus- Petals | | | | | | |
| --- | --- | --- | --- | --- | --- | --- |
| Compound | ZNH | KF | PLD | LS | ZQ | YMY |
| 3-Thujene | 6.16±2.43 | 12.23±2.67 | 30.92±5.73 | 19.54±7.12 | 20.79±3.16 | 21.38±1.83 |
| α-Pinene | 26.73±10.99 | 52.74±13.87 | 134.76±31.4 | 73.82±27.11 | 95.15±3.27 | 78.34±5.43 |
| Camphene | - | - | 1.84±0.75 | - | - | - |
| Sabinen | 82.95±33.87 | 209.58±30.4 | 420.19±152.7 | 239±82 | 402.14±106.87 | 316.9±96.01 |
| β-Pinene | 11.73±4.97 | 23.52±5.62 | 59.57±13.72 | 32.23±11.14 | 42.87±1.72 | 33.82±2.97 |
| β-Myrcene | 36.37±14.76 | 71.49±22.66 | 172.09±46.17 | 92.31±27.83 | 125.3±4.88 | 96.96±11.38 |
| α-Phellandrene | - | - | 2.45±0.4 | - | 1.33±0.07 | - |
| α-Terpinene | 33.67±13.75 | 58.48±21.41 | 166.54±31.84 | 93.71±27.59 | 95.9±20.38 | 79.73±7.79 |
| *D*-Limonene | 47.08±19.87 | 92.14±24.52 | 228.02±56.33 | 125.97±35.46 | 162.94±3.01 | 133.72±15.17 |
| Eucalyptol | 109.78±40.23 | 209.79±37.86 | 463.96±77.96 | 249.13±66.1 | 392.81±32.29 | 313.8±38.14 |
| β-*cis*-Ocimene | - | - | 3.57±1.06 | - | 3.01±0.52 | 1.88±0.31 |
| γ-Terpinene | 64.65±26.35 | 114±34.95 | 302.6±48.49 | 169.83±48.81 | 211.05±50.2 | 170.5±10.06 |
| Terpinolene | 14.87±6.62 | 28.99±8.79 | 74.4±12.2 | 39.48±12.32 | 60.85±12.64 | 47.56±3.5 |
| Terpinen-4-ol | 67.7±25.62 | 131.71±55.39 | 238.63±42.15 | 155.75±26.29 | 496.42±144.73 | 354.85±34.88 |
| α-Terpineol | 30.49±14.41 | 54.5±16.2 | 78.03±13.91 | 54.99±9.66 | 236.89±71.59 | 122.77±4.35 |
| Caryophyllene | 684.91±123.74 | 507.82±254.94 | - | 250.82±118.23 | 15.55±0.6 | 184.67±42.42 |
| Humulene | 85.92±13.42 | 64.15±29.71 | - | 34.33±13.13 | - | 27.99±4.38 |
| γ-Muurolene | - | - | - | - | 16.5±1.91 | 14.41±0.23 |
| Germacrene D | 23.16±3.71 | 45.81±14.67 | 39.91±2.29 | - | 60.5±22.04 | 35.25±5.77 |
| δ-Cadinene | 15.83±2.54 | - | - | - | - | - |
| Caryophyllene oxide | - | - | 105.5±13.22 | - | - | - |
| γ-Eudesmol | - | 19.48±2.63 | 15.58±1.68 | 48.16±12.75 | 21.43±4.49 | 30.91±8.25 |
| o-Cymene | 65.4±1.82 | 67.97±2.27 | 76.97±1.2 | 71.4±1.34 | 72.3±3.04 | 68.51±0.87 |
| 1,4-Dimethoxybenzene | 850.68±110.5 | 1279.16±169.98 | 1079.39±147.23 | 830.37±74.31 | 803.55±128.24 | 1184.88±63.03 |
| Table S2 (*continued*) | | | | | | |
| Compound | ZNH | KF | PLD | LS | ZQ | YMY |
| Undecane | - | 10.99±1.19 | 9.8±0.08 | 11.33±1.06 | 10.79±1.04 | 11.48±0.59 |
| Dodecane | - | - | - | 10.11±0.56 | 10.03±0.56 | 9.72±0.29 |
| (6*E*)-6-Tridecene | 10.51±0.53 | - | 9.54±0.03 | 10.23±0.44 | 10.95±0.51 | 10.5±0.22 |
| Tridecane | 35.98±4.18 | 30.58±7.04 | 33.06±1.01 | 39.86±0.27 | 41.5±6.09 | 46.4±6.23 |
| (*E*)-4-Tetradecene | 13.52±0.85 | - | 11.87±0.4 | 13.13±1.05 | 19.58±2.08 | 14.89±0.79 |
| 1-Tetradecene | 16.17±1.08 | 13.67±1.23 | 11.94±0.14 | 14.01±0.79 | 16.9±1.72 | 13.09±0.36 |
| Jasmone | - | - | - | - | - | 10.26±1.3 |
| Tetradecane | 85.28±13.33 | 70.77±14.76 | 79.01±4.74 | 63.89±2.74 | 116.79±17.32 | 97.32±12.18 |
| 1-Pentadecene | 1015.46±84.71 | 642.85±133.33 | 620.89±96.34 | 790.9±79.38 | 1369.59±91.19 | 1060.41±173.7 |
| Pentadecane | 3673.18±587.61 | 2663.39±369.54 | 2256.83±415.98 | 1940.79±125.24 | 3656.77±226.74 | 3600.78±394.11 |
| (*Z*)-7-Hexadecene | 51.53±8.91 | - | - | 27.31±2.72 | 63.77±12.5 | 32.53±1.17 |
| (*Z*)-3-Hexadecene | - | - | 14.61±0.87 | - | - | - |
| Cetene | 20.85±2.21 | 16.13±0.75 | 14.93±1.03 | 15.15±1.4 | 25.05±4.74 | 14.32±0.83 |
| Hexadecane | 44.27±6.18 | 33.13±3.17 | 24.04±3.35 | 29.29±3.91 | 42.63±8.87 | 27.84±1.74 |
| 6,9-Heptadecadiene | 1197.28±250.44 | 865.14±176.45 | 592.82±108.82 | 689.81±45.72 | 1194.72±250.65 | 718.07±62.97 |
| 1-Tetradecanol | 741.49±219.47 | 647.33±148.68 | 466.94±86.4 | 410.79±30.24 | 997.35±132.74 | 701.82±18.12 |
| 8-Heptadecene | 263.81±17.21 | 157.15±30.51 | 158.89±50.05 | 165.74±31.44 | 346.69±57.08 | 260.38±82.32 |
| Heptadecane | 226.19±30.99 | 164.86±18.34 | 108.63±23.86 | 141.61±16.32 | 189.9±35.26 | 153.37±20.48 |
| Note: All concentrations are expressed in ng/g/h FW; We use abbreviations instead of ancient lotus species names, e.g. ZNH stands for *N. nucifera* ‘Zhongnanhai Antique’ (this nomenclature rule has been mentioned in the main text). “-” means not detected. | | | | | | |
