# Supplementary material for: Untargeted metabolites profiling of volatile components of Chinese Antique Lotus (Nelumbo nucifera Gaertn.) using solid-phase microextraction (SPME) GC/MS
Source: PeerJ. 2025 Jun 19;13:e19600. doi: 10.7717/peerj.19600 (PMC12182725; doi:10.7717/peerj.19600)
Supplement: Supplemental Information 3 — Note: we use abbreviations instead of ancient lotus species names, e.g. ZNH stands for N. nucifera ‘Zhongnanhai Antique’ (this nomenclature rule has been mentioned in the main text). “ -” means not detected. [file peerj-13-19600-s003.docx]

| Table S3. Compound content in each tissue of antique lotus- Stamens | | | | | | | | | |
| --- | --- | --- | --- | --- | --- | --- | --- | --- | --- |
| Compound | ZNH | | KF | | PLD | LS | ZQ | YMY |  |
| 3-Thujene | 2.64±0.73 | | 3.88±1.63 | | 10.46±2.01 | 4.62±1.38 | 12.88±0.48 | 4.87±1.3 |  |
| α-Pinene | 11.37±2.68 | | 22.93±10.07 | | 45.87±9.32 | 22.58±5 | 59.9±0.7 | 33.79±6.83 |  |
| Sabinen | 43.67±14.21 | | 59.68±18.12 | | 169.89±42.91 | 67.12±15.15 | 202.56±7.1 | 93.45±34.06 |  |
| β-Pinene | 6.75±1.7 | | 15.23±6.67 | | 25.66±5.38 | 14.45±3.35 | 39.05±0.57 | 24.28±4.11 |  |
| β-Myrcene | 22.64±2.13 | | 61.11±18.66 | | 57.39±18.09 | 32.16±9.13 | 104.6±3.49 | 64.46±15.04 |  |
| (+)-2-Carene | 76.1±8.45 | | 117.58±8.11 | | 52.8±8.25 | 98.36±19.69 | 44.25±1.01 | 30.25±0.5 |  |
| α-Phellandrene | - | | - | | 1.47±0.03 | - | 2.7±0.37 | - |  |
| α-Terpinene | 29.64±7.78 | | 80.54±38.44 | | 89.24±19.9 | 72.26±19.98 | 174.34±11.61 | 111.53±15.27 |  |
| D-Limonene | 47.15±11.67 | | 107.39±44.95 | | 121.93±27.64 | 94.64±23.52 | 238.75±14.09 | 150.54±21.16 |  |
| Eucalyptol | 100.83±24.48 | | 221.64±92.01 | | 317.53±63.78 | 203.99±25.22 | 495.45±14.78 | 374.39±53.62 |  |
| β-cis-Ocimene | - | | - | | - | - | 2.45±0.14 | 1.78±0.55 |  |
| γ-Terpinene | 73.42±19.9 | | 180.26±81.2 | | 211.33±51.49 | 168.95±36.79 | 407.28±22.82 | 287.38±38.59 |  |
| Terpinolene | 20.13±5 | | 47.63±21.07 | | 53.5±13.89 | 41.75±9.05 | 106.82±4.8 | 74.53±11.33 |  |
| cis-2-p-Menthen-1-ol | 12.62±1.19 | | 16.5±2.5 | | 10.69±2.42 | 15.15±3.39 | 8.37±0.2 | 5.9±1.24 |  |
| Terpinen-4-ol | 19.34±5.91 | | 66.98±33.96 | | 108.68±29.71 | 72.93±9.46 | 167.54±8.2 | 167.68±33.86 |  |
| α-Terpineol | 1.68±0.83 | | 6.97±2.49 | | 7.51±2.76 | 6.95±0.71 | 20.32±0.86 | 15.63±5.63 |  |
| α-Cyclogeraniol | 13.81±4.3 | | - | | 28.86±30.81 | 14±1.83 | 5.27±3.87 | 38.83±24.33 |  |
| Copaene | 20.6±1.11 | | - | | - | - | - | 17.28±1.53 |  |
| (-)-β-Elemene | 17.11±0.59 | | - | | - | - | - | - |  |
| Caryophyllene | 3052.22±330.71 | | 2992.77±700.75 | | - | - | - | 1575.23±267.24 |  |
| cis-β-Farnesene | 21.13±0.57 | | 21.96±2.66 | | - | 38.93±19.99 | - | 27.74±13.4 |  |
| Humulene | 401.01±50.51 | | 388.49±105.4 | | - | 157.5±31.68 | - | 187.82±30.27 |  |
| γ-Gurjunene | - | | 26.02±4.6 | | - | 15.56±1.28 | - | 16.67±0.82 |  |
| γ-Muurolene | 16.39±1.2 | | - | | - | 13.25±0.3 | - | 13.5±0.64 |  |
| Table S3 (*continued*) | | | | | | | | | |
| Compound | ZNH | KF | | PLD | | LS | ZQ | YMY |  |
| Germacrene D | 92.27±10.41 | 90.06±29.78 | | - | | 38.72±8.05 | - | 46.34±6.8 |  |
| (+)-Bicyclogermacrene | 33.56±3.49 | 29.34±9.51 | | - | | 18.28±1.22 | - | 17.78±1.11 |  |
| δ-Guaiene | 23.71±1.87 | 25.25±6.09 | | - | | - | - | 17.05±2.12 |  |
| γ-Cadinene | 23.54±1.94 | 23.22±4.39 | | - | | 14.82±1.08 | - | 15.63±0.71 |  |
| δ-Cadinene | 51.27±6.61 | 49.74±13.66 | | - | | 23.4±3.9 | - | 26.57±2.54 |  |
| α-Cadinene | 14.36±0.8 | - | | - | | - | - | - |  |
| Caryophyllene oxide | 181.11±43.36 | 197.66±108.18 | | - | | 37.24±20.93 | - | 60.87±15.29 |  |
| γ-Eudesmol | 18.06±1.19 | 29.16±4.88 | | 18.77±3 | | 32.54±4.89 | 26.46±2.1 | 47.28±7.45 |  |
| α-epi-Cadinol | 21.42±3.21 | - | | - | | - | - | 14.82±1.15 |  |
| α-Eudesmol | - | - | | - | | - | - | 15.35±1.06 |  |
| α-Cadinol | 24.33±2.99 | 28.96±6.07 | | - | | - | - | 14.16±0.79 |  |
| o-Cymene | 77.36±4.47 | 88.08±11.92 | | 84.75±4.45 | | 83.52±7.75 | 115.69±3.84 | 90.32±2.69 |  |
| 1,4-Dimethoxybenzene | 2602.98±124.08 | 3646.52±428.69 | | 3494.67±416.92 | | 2753.75±292.16 | 3041.1±461.52 | 5173.32±268.64 |  |
| 1-Hexanol | 9.52±0.28 | 9.81±0.61 | | - | | 9.81±0.29 | 9.9±0.48 | 9.79±0.53 |  |
| 1-Butanol, 3-methyl-, acetate | 9.16±0.15 | - | | - | | - | - | - |  |
| 1-Cyclohexene-1-carboxylic acid, 2,6,6-trimethyl-, methyl ester | - | - | | - | | - | - | 9.01±0.07 |  |
| (6E)-6-Tridecene | - | 11.54±0.13 | | - | | - | 8.94±0.01 | - |  |
| Tridecane | 11.13±0.31 | - | | 11.54±0.3 | | 11.4±0.26 | 11.94±0.75 | 13.23±1.2 |  |
| (E)-4-Tetradecene | - | - | | - | | - | 8.94±0.03 | - |  |
| 1-Tetradecene | 9.66±0.15 | - | | 9.06±0.16 | | 9.31±0.17 | 9.83±0.08 | 9.79±0.51 |  |
| Jasmone | 18.61±3.41 | 41.56±21.69 | | 16.04±3.82 | | 73.77±45.8 | 27.14±7.82 | 29.93±0.65 |  |
| Tetradecane | 12.77±0.76 | 14.84±1.31 | | 13.89±1.51 | | 14.06±0.76 | 15.71±0.76 | 15.3±2.45 |  |
| Table S3 (*continued*) | | | | | | | | | |
| Compound | ZNH | KF | | PLD | | LS | ZQ | YMY |  |
| 1-Pentadecene | 36.17±3.34 | 46.24±0.35 | | 39.12±12.95 | | 44.01±16.59 | 76.4±0.62 | 59.43±20.08 |  |
| Pentadecane | 105.59±19.23 | 147.82±9.05 | | 100.49±25.44 | | 87.68±31.59 | 177.49±3.12 | 166.06±61.99 |  |
| (Z)-7-Hexadecene | - | - | | - | | - | 9.82±0.22 | - |  |
| Cetene | - | - | | - | | - | 9.13±0.05 | - |  |
| Hexadecane | 9.36±0.19 | - | | - | | - | 9.58±0.13 | 9.23±0.33 |  |
| 6,9-Heptadecadiene | 27.99±5.62 | 38.26±7.75 | | 18.54±2.69 | | 22.36±7.95 | 31.51±6.9 | 23.11±6.62 |  |
| 1-Tetradecanol | 35.66±7.09 | 54.44±10.38 | | 27.82±2.27 | | 28.74±10.38 | 52.7±9.5 | 40.62±12.85 |  |
| 8-Heptadecene | 25.76±2.78 | 27.87±3.55 | | 18.88±4.01 | | 21.3±6.19 | 35.43±5.39 | 26.41±4.66 |  |
| Heptadecane | 19.31±2.04 | 22.29±2.12 | | 13.99±0.9 | | 16.58±2.79 | 20.02±2.24 | 19.14±2.92 |  |
| Note: All concentrations are expressed in ng/g/h FW; We use abbreviations instead of ancient lotus species names, e.g. ZNH stands for *N. nucifera* ‘Zhongnanhai Antique’ (this nomenclature rule has been mentioned in the main text). “-” means not detected. | | | | | | | | | |
