# Supplementary material for: Untargeted metabolites profiling of volatile components of Chinese Antique Lotus (Nelumbo nucifera Gaertn.) using solid-phase microextraction (SPME) GC/MS
Source: PeerJ. 2025 Jun 19;13:e19600. doi: 10.7717/peerj.19600 (PMC12182725; doi:10.7717/peerj.19600)
Supplement: Supplemental Information 4 — Note: we use abbreviations instead of ancient lotus species names, e.g. ZNH stands for N. nucifera ‘Zhongnanhai Antique’ (this nomenclature rule has been mentioned in the main text). “ -” means not detected. [file peerj-13-19600-s004.docx]

| Table S4. Compound content in each tissue of antique lotus- Receptacle | | | | | | | |
| --- | --- | --- | --- | --- | --- | --- | --- |
| Compound | ZNH | KF | PLD | LS | ZQ | YMY | |
| 3-Thujene | - | - | - | - | 2.01±0.06 | - | |
| α-Pinene | 4.98±1.66 | 5.02±1.91 | 18.92±5.5 | 7.16±2.79 | 12.62±0.45 | 7.64±1.42 | |
| Sabinen | 2.94±0.87 | 2.4±0.58 | - | 5.73±3.9 | 17.65±1.81 | 6.87±6.35 | |
| β-Pinene | 2.62±0.95 | 3.52±1.42 | 10.47±3.12 | 4.22±1.26 | 9.51±0.2 | 6.43±1.16 | |
| β-Myrcene | - | - | 18.03±8.67 | - | 14.46±1.61 | 9.18±2.21 | |
| (+)-2-Carene | 26.41±5.82 | 38.54±2.2 | 28.51±7.73 | 35±1.95 | 26.52±0.83 | 23.52±9.08 | |
| α-Phellandrene | - | - | 2.37±1.07 | - | - | - | |
| α-Terpinene | 10.45±4.34 | 25.65±12.2 | 89.5±33.76 | 27.55±8.05 | 61.12±1.75 | 55±11.23 | |
| D-Limonene | 13.84±5.48 | 28.28±12.71 | 80.38±29.8 | 25.94±6.73 | 72.44±2.65 | 59.41±10.33 | |
| Eucalyptol | 113.86±29.91 | 223.93±84.21 | 382.22±97.23 | 233.34±70.25 | 492.89±32.64 | 420.33±72.04 | |
| γ-Terpinene | 33.52±12.42 | 73.22±33.6 | 220.34±83.05 | 78.52±20.3 | 191.9±9.64 | 165.2±26.52 | |
| Terpinolene | 8.41±3.52 | 18.98±9.2 | 57.16±23.42 | 19.74±5.44 | 51.6±2.81 | 43.2±7.21 | |
| cis-2-p-Menthen-1-ol | - | 1.53±0.38 | - | - | - | - | |
| Terpinen-4-ol | 13.95±3.57 | 57.91±32.6 | 95.71±59.58 | 93.85±47.85 | 168.78±27.4 | 156.3±28.96 | |
| α-Terpineol | - | 5.87±3.55 | 7.73±6.39 | 12.27±6.67 | 17.94±4.99 | 9.83±1.59 | |
| α-Cyclogeraniol | - | 4.13±0.87 | - | 5.9±3.83 | 2.61±1.09 | 6.19±2.38 | |
| Caryophyllene | 380.68±80.27 | 478.36±46.06 | 14.14±0.64 | 218.44±46.72 | 12.75±0.32 | 165.1±28.59 | |
| cis-β-Farnesene | - | 14.01±0.53 | - | - | - | - | |
| Humulene | 51.08±8.86 | 63.77±5.18 | - | 32.54±4.65 | - | 27.21±3.35 | |
| (E)-β-Famesene | 14.01±0.5 | - | - | 14.11±0.58 | - | 14.01±0.18 | |
| Germacrene D | 15.45±1.31 | 17.67±1 | - | - | - | - | |
| δ-Selinene | 16.1±0.61 | 16.08±1.62 | 15.38±0.51 | - | - | 14.77±0.52 | |
| δ-Guaiene | - | 16.12±0.77 | - | - | - | - | |
| δ-Cadinene | 14.71±0.76 | - | - | 13.09±0.17 | - | - | |
| Table S4 (*continued*) | | | | | | |  |
| Compound | ZNH | KF | PLD | LS | ZQ | YMY | |
| Caryophyllene oxide | - | 15.72±1.2 | - | - | - | - | |
| γ-Eudesmol | 24.58±12.29 | 53.13±13.3 | 28.61±2.62 | 50±6.27 | 36.25±14.12 | 23.18±5.88 | |
| α-epi-Cadinol | - | 22.18±5.55 | - | - | - | - | |
| α-Eudesmol | - | 23.7±7.58 | - | - | - | - | |
| o-Cymene | 73.61±4.71 | 77.49±6.76 | 97.08±9.26 | 80.13±5.57 | 93.4±4.38 | 89.02±4.98 | |
| 1,4-Dimethoxybenzene | 1010.88±123.9 | 1434.69±148.19 | 1627.26±255.31 | 1188.3±96.71 | 1418.7±297.96 | 1619.81±168.46 | |
| Butanoic acid, 2-methyl-, ethyl ester | - | - | - | 9.22±0.11 | - | - | |
| 1-Hexanol | 12.34±2.66 | 11.37±0.84 | 9.08±0.15 | 14.66±3.14 | 15.41±2.58 | 16.94±1.49 | |
| Butyric acid, 2-hydroxy-3-methyl-, methyl ester | - | - | - | - | 9.07±0.15 | - | |
| Nonane | - | 10.63±0.48 | - | - | 9.54±0.5 | 9.41±0.51 | |
| 1-Cyclohexene-1-carboxylic acid, 2,6,6-trimethyl-, methyl ester | - | 9.41±0.23 | - | 12.22±1.24 | 11.12±0.91 | - | |
| Tridecane | 10.45±0.29 | 10.58±0.31 | 10.82±0.42 | 11.21±0.53 | 11.94±0.19 | 12.17±0.66 | |
| 1-Tetradecene | - | - | - | - | 9.48±0.11 | - | |
| Jasmone | - | - | - | - | - | 9.55±0.53 | |
| Tetradecane | 10.4±0.32 | 10.6±0.38 | 10.74±0.38 | 10.37±0.65 | 13.04±0.53 | 11.09±0.56 | |
| 1-Pentadecene | 19.35±1.41 | 19.13±1.63 | 17.27±1.22 | 17.9±3.26 | 39.8±4.16 | 22.24±6.09 | |
| Pentadecane | 43.83±8.42 | 40.62±3.87 | 35.61±2.54 | 28.31±6.3 | 73.37±5.21 | 55.84±26.88 | |
| 6,9-Heptadecadiene | 14.05±3.4 | 15.43±1.59 | 11.56±0.31 | 11.85±1.05 | 17.85±1.85 | 14.36±5.93 | |
| 1-Tetradecanol | 18.56±4.75 | 20.52±1.9 | 16.07±0.88 | 15.04±2.14 | 29.12±3.16 | 22.05±11.28 | |
| 8-Heptadecene | 12.99±1.88 | 12.39±0.46 | 11.44±0.61 | 11.63±0.86 | 17.39±1.34 | 13.28±3.08 | |
| Heptadecane | 11.84±1.33 | 11.8±0.45 | 10.29±0.31 | 10.96±0.5 | 13.25±0.71 | 11.99±2.31 | |
| Note: All concentrations are expressed in ng/g/h FW; We use abbreviations instead of ancient lotus species names, e.g. ZNH stands for *N. nucifera* ‘Zhongnanhai Antique’ (this nomenclature rule has been mentioned in the main text). “-” means not detected. | | | | | | | |
