# Supplementary material for: Untargeted metabolites profiling of volatile components of Chinese Antique Lotus (Nelumbo nucifera Gaertn.) using solid-phase microextraction (SPME) GC/MS
Source: PeerJ. 2025 Jun 19;13:e19600. doi: 10.7717/peerj.19600 (PMC12182725; doi:10.7717/peerj.19600)
Supplement: Supplemental Information 5 [file peerj-13-19600-s005.docx]

**Table S5 rOAV of petals from different Antique Lotus cultivars**

| No. | CAS | Compound | Odor detection threshold (μg/kg) | OAV | | | | | | Odor description |
| --- | --- | --- | --- | --- | --- | --- | --- | --- | --- | --- |
|  |  |  |  | ZNH | KF | PLD | LS | ZQ | YMY |  |
| 1 | 7785-70-8 | α-Pinene | 2.2 | 12.15±4.99 | 23.97±6.31 | 61.25±14.27 | 33.56±12.32 | 43.25±1.48 | 35.61±2.47 | Fresh，woody |
| 2 | 123-35-3 | β-Myrcene | 1.2 | 30.3±12.3 | 59.57±18.89 | 143.41±38.47 | 76.92±23.19 | 104.42±4.07 | 80.8±9.48 | Sweet, spicy, plastic |
| 3 | 99-86-5 | α-Terpinene | 80 | 0.42±0.17 | 0.73±0.27 | 2.08±0.4 | 1.17±0.34 | 1.2±0.25 | 1±0.1 | Citrusy, herbal, woody |
| 4 | 5989-27-5 | Limonene | 34 | 1.38±0.58 | 2.71±0.72 | 6.71±1.66 | 3.71±1.04 | 4.79±0.09 | 3.93±0.45 | Citrusy, pine scent, peppermint |
| 5 | 470-82-6 | Eucalyptol | 1.1 | 99.8±36.58 | 190.72±34.42 | 421.78±70.88 | 226.48±60.09 | 357.1±29.35 | 285.27±34.67 | Camphoraceous, fresh, grassy |
| 6 | 87-44-5 | Caryophyllene | 64 | 10.7±1.93 | 7.93±3.98 | 0±0 | 3.92±1.85 | 0.24±0.01 | 2.89±0.66 | Woody, spicy, clove flower fragrance |
