# Supplementary material for: Untargeted metabolites profiling of volatile components of Chinese Antique Lotus (Nelumbo nucifera Gaertn.) using solid-phase microextraction (SPME) GC/MS
Source: PeerJ. 2025 Jun 19;13:e19600. doi: 10.7717/peerj.19600 (PMC12182725; doi:10.7717/peerj.19600)
Supplement: Supplemental Information 6 [file peerj-13-19600-s006.docx]

Table S6 rOAV of stamens from different Antique Lotus cultivars

| No. | CAS | Compound | Odor detection threshold (μg/kg) | OAV | | | | | | Odor description |
| --- | --- | --- | --- | --- | --- | --- | --- | --- | --- | --- |
|  |  |  |  | ZNH | KF | PLD | LS | ZQ | YMY |  |
| 1 | 7785-70-8 | α-Pinene | 2.2 | 5.17±1.22 | 10.42±4.58 | 20.85±4.24 | 10.26±2.27 | 27.23±0.32 | 15.36±3.1 | Fresh，woody |
| 2 | 123-35-3 | β-Myrcene | 1.2 | 18.86±1.78 | 50.92±15.55 | 47.83±15.08 | 26.8±7.6 | 87.16±2.91 | 53.72±12.53 | Sweet, spicy, plastic |
| 3 | 99-86-5 | α-Terpinene | 80 | 0.37±0.1 | 1.01±0.48 | 1.12±0.25 | 0.9±0.25 | 2.18±0.15 | 1.39±0.19 | Citrusy, herbal, woody |
| 4 | 5989-27-5 | Limonene | 34 | 1.39±0.34 | 3.16±1.32 | 3.59±0.81 | 2.78±0.69 | 7.02±0.41 | 4.43±0.62 | Citrusy, pine scent, peppermint |
| 5 | 470-82-6 | Eucalyptol | 1.1 | 91.67±22.25 | 201.49±83.65 | 288.66±57.98 | 185.44±22.93 | 450.41±13.43 | 340.35±48.75 | Camphoraceous, fresh, grassy |
| 6 | 87-44-5 | Caryophyllene | 64 | 47.69±5.17 | 46.76±10.95 | 0±0 | 21.48±3.53 | 0±0 | 24.61±4.18 | Woody, spicy, clove flower fragrance |
| 7 | 6753-98-6 | Humulene | 160 | 2.51±0.32 | 2.43±0.66 | 0±0 | 0.98±0.2 | 0±0 | 1.17±0.19 | Woody |
| 8 | 111-27-3 | 1-Hexanol | 5.6 | 1.7±0.05 | 1.75±0.11 | 0±0 | 1.75±0.05 | 1.77±0.09 | 1.75±0.1 | Grass, wood |
