# Supplementary material for: Untargeted metabolites profiling of volatile components of Chinese Antique Lotus (Nelumbo nucifera Gaertn.) using solid-phase microextraction (SPME) GC/MS
Source: PeerJ. 2025 Jun 19;13:e19600. doi: 10.7717/peerj.19600 (PMC12182725; doi:10.7717/peerj.19600)
Supplement: Supplemental Information 7 [file peerj-13-19600-s007.docx]

Table S7 rOAV of receptacle from different Antique Lotus cultivars

| No. | CAS | Compound | Odor detection threshold (μg/kg) | OAV | | | | | | Odor description |
| --- | --- | --- | --- | --- | --- | --- | --- | --- | --- | --- |
|  |  |  |  | ZNH | KF | PLD | LS | ZQ | YMY |  |
| 1 | 7785-70-8 | α-Pinene | 2.2 | 2.26±0.75 | 2.28±0.87 | 8.6±2.5 | 3.25±1.27 | 5.73±0.21 | 3.47±0.65 | Fresh，woody |
| 2 | 123-35-3 | β-Myrcene | 1.2 | 0±0 | 0±0 | 15.02±7.23 | 0±0 | 12.05±1.34 | 7.65±1.84 | Sweet, spicy, plastic |
| 3 | 99-86-5 | α-Terpinene | 80 | 0.13±0.05 | 0.32±0.15 | 1.12±0.42 | 0.34±0.1 | 0.76±0.02 | 0.69±0.14 | Citrusy, herbal, woody |
| 4 | 5989-27-5 | Limonene | 34 | 0.41±0.16 | 0.83±0.37 | 2.36±0.88 | 0.76±0.2 | 2.13±0.08 | 1.75±0.3 | Citrusy, pine scent, peppermint |
| 5 | 470-82-6 | Eucalyptol | 1.1 | 103.51±27.19 | 203.57±76.56 | 347.48±88.39 | 212.13±63.86 | 448.08±29.68 | 382.11±65.49 | Camphoraceous, fresh, grassy |
| 6 | 87-44-5 | Caryophyllene | 64 | 5.95±1.25 | 7.47±0.72 | 0.22±0.01 | 3.41±0.73 | 0.2±0 | 2.58±0.45 | Woody, spicy, clove flower fragrance |
| 7 | 111-27-3 | 1-Hexanol | 5.6 | 2.2±0.47 | 2.03±0.15 | 1.62±0.03 | 2.62±0.56 | 2.75±0.46 | 3.02±0.27 | Grass, wood |
