# Supplementary figures and images for: Untargeted metabolites profiling of volatile components of Chinese Antique Lotus (Nelumbo nucifera Gaertn.) using solid-phase microextraction (SPME) GC/MS
Source: PeerJ. 2025 Jun 19;13:e19600. doi: 10.7717/peerj.19600 (PMC12182725; doi:10.7717/peerj.19600)

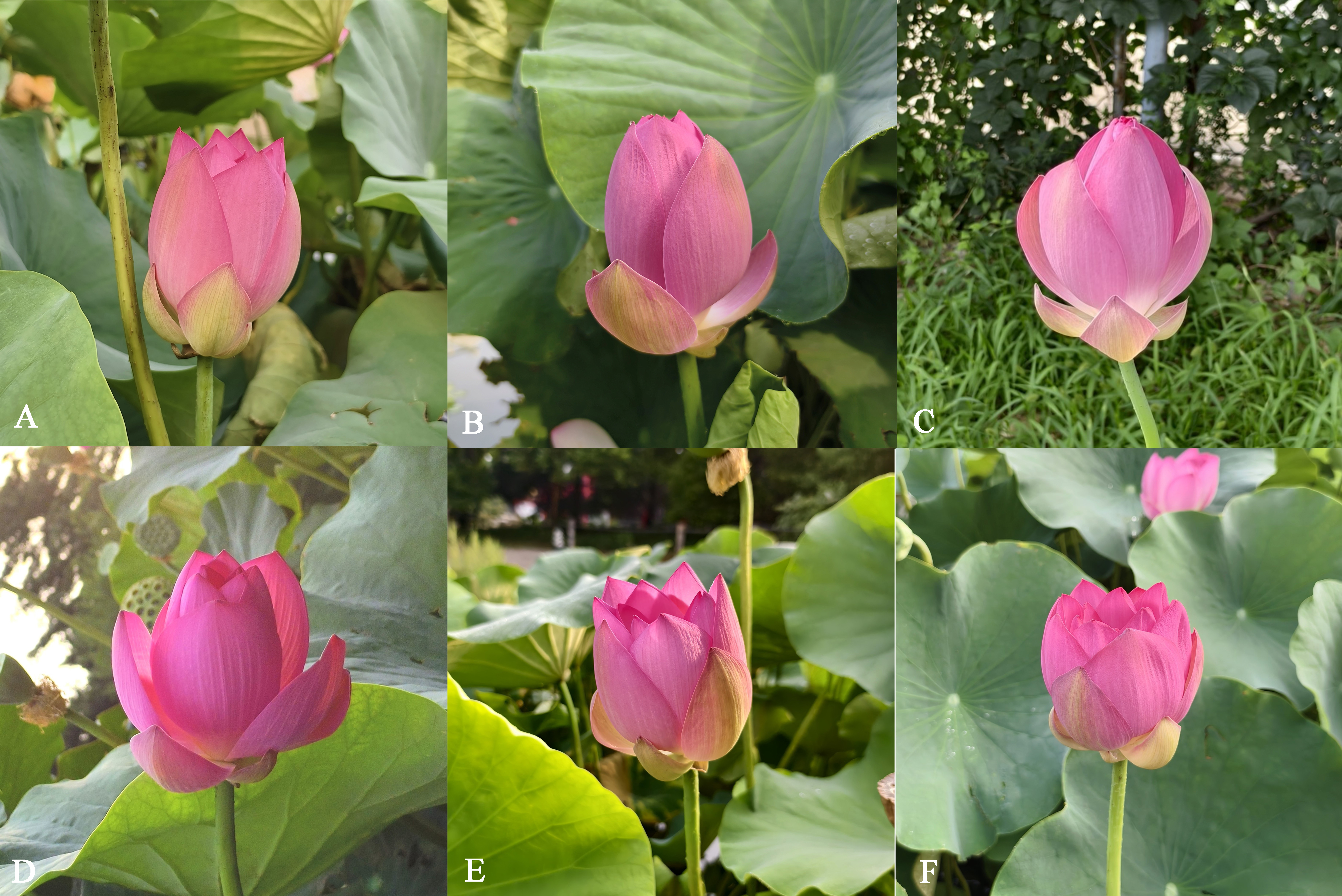

Supplement: Supplemental Information 8 — (a) ZNH; (b)KF; (c)PLD; (d)LS; (e)ZQ; (f)YMY [file peerj-13-19600-s008.jpg]

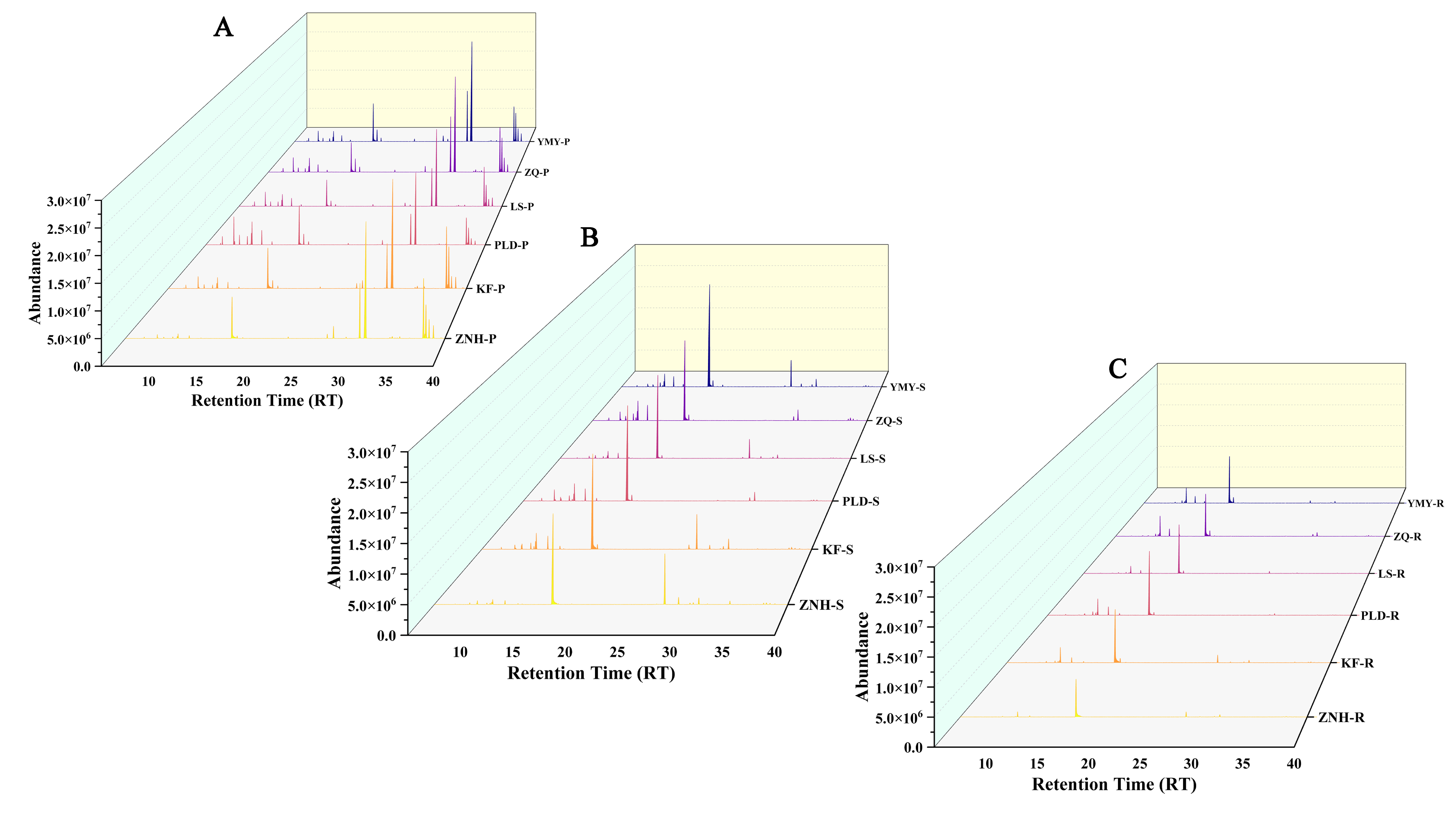

Supplement: Supplemental Information 9 — (A) petals; (B) stamens; (C) receptacles. ZNH, KF, PLD, LS, ZQ, and YMY indicate Antique Lotus. P, S, and R indicate petals, stamens, and receptacles respectively. Such as, ZNH-P is the organization of the petals of ZNH. [file peerj-13-19600-s009.jpg]

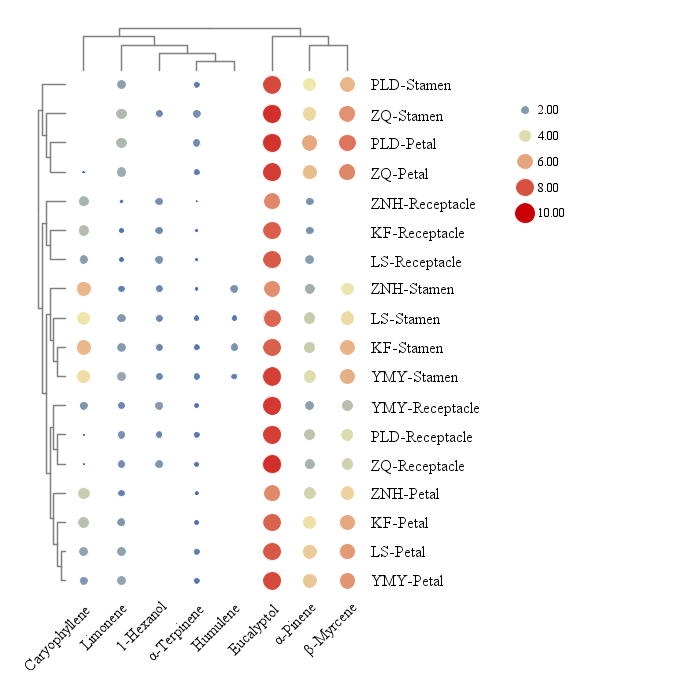

Supplement: Supplemental Information 10 [file peerj-13-19600-s010.png]

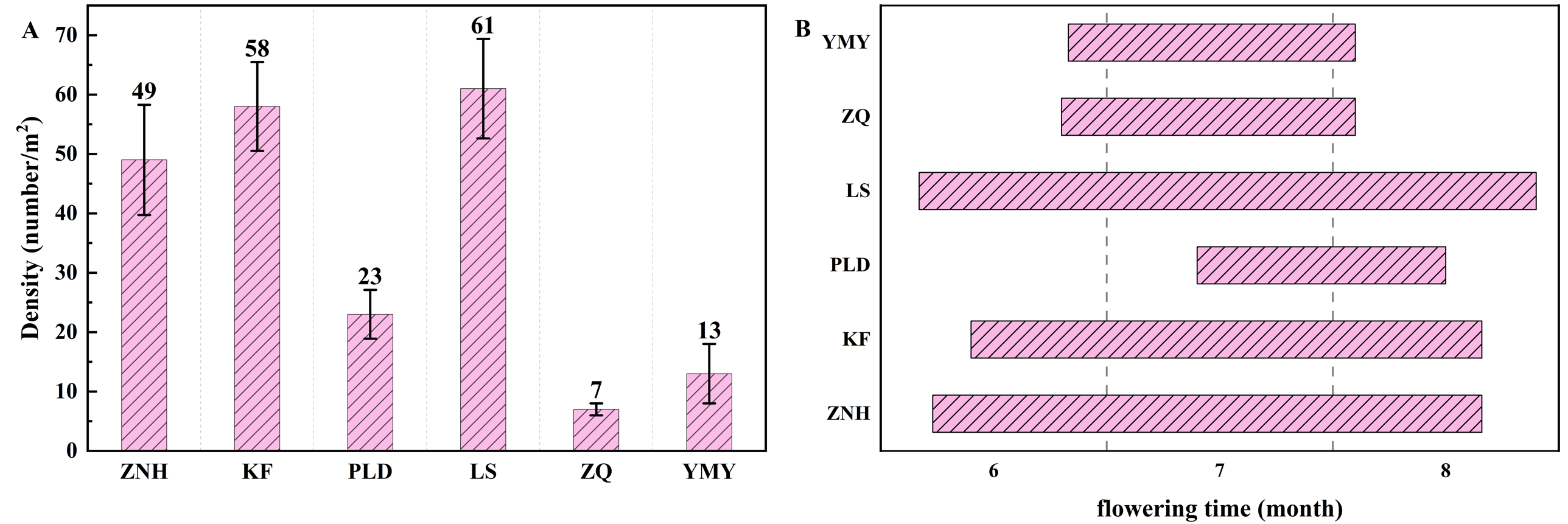

Supplement: Supplemental Information 11 — (A) Histogram of the number of flowers per square metre for the 6 Antique Lotus; (B) Comparison of the length of the flowering period of 6 Ancient Lotus flowers. [file peerj-13-19600-s011.jpg]
